# Family medicine and primary health care in Liberia

**DOI:** 10.4102/phcfm.v18i1.5402

**Published:** 2026-05-05

**Authors:** David Okiror, Ibrahim Sanoe

**Affiliations:** 1 School of Medicine, College of Health Sciences, University of Liberia, Monrovia, Liberia; 2 College of Physicians, Faculty of Family Medicine, Liberia College of Physicians and Surgeons, Monrovia, Liberia; 3 General Outpatient Clinic, John F. Kennedy Medical Center, Monrovia, Liberia; 4 Society of Family Physicians of Liberia, Department of Health Education and Delivery, Monrova, Liberia; 5 Ministry of Health, Department of Curative Services, Monrovia, Liberia

**Keywords:** Liberia, profile, family, medicine, training

## Abstract

This country profile examines the development of family medicine (FM) and primary health care (PHC) in Liberia, a low-income West African nation with significant health and socioeconomic challenges. Liberia’s PHC system is structured across three tiers and relies heavily on community health workers to serve dispersed and underserved populations. Family medicine is an emerging specialty, crucial for strengthening PHC and advancing universal health coverage, especially in rural areas facing workforce shortages. The Family Medicine Specialty Training Program, launched in 2017, addresses the deficit of rural healthcare providers through comprehensive, context-specific education and rural rotations. Family physicians contribute at multiple levels of the health system, providing clinical care, mentorship, and leadership. The Society of Family Physicians of Liberia supports professional development and advocacy. Despite ongoing challenges, Liberia’s integration of FM and PHC, supported by national and international partnerships, demonstrates significant progress and offers valuable lessons for building resilient health systems in low-resource settings.

## Introduction

**FIGURE 1:**
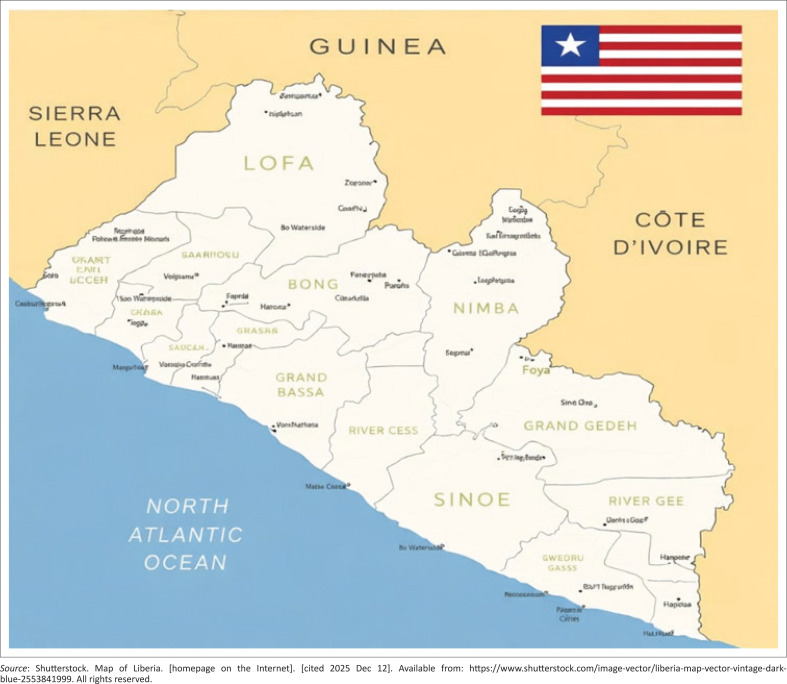
Map of Liberia.^3^

Liberia lies in West Africa and is bordered by Sierra Leone to the west, Guinea to the north, Côte d’Ivoire to the east, and the Atlantic Ocean to the south (Figure 1). The country has a land size of 111 369 km^2^ and a coastline that is up to 580 km long.^1,2^

Liberia is a parliamentary representative democratic republic, and the president is the head of government. The government has executive power, and both the government and parliament have legislative power. The government has both central and decentralised levels of local government.

There are 5.2 million people in the country, with 50.4% of them being men and 49.6% being women.^1^ Liberia is one of the least populated countries in the world, with an estimated population density of 45 people per square kilometre.

Liberia has a wide range of religions. About 85.6% of the people are Christians, 12.6% are Muslims, and 1.2% are from traditional or other faiths.^1^ Urban cities have grown substantially because of population growth and internal migration from rural areas. Because of this, urban slums are underserved and overcrowded, and many families have lost their social support networks. A large section of the population is young, with 43.4% being under 15 years. Many young people will reach reproductive age in the next few decades and may struggle with challenges such as drug misuse and teenage pregnancies.

Liberia is a low-income country. In 2020, the country’s gross national income was $633.00 per person in terms of purchasing power parity.^1^ The gross domestic product (GDP) fell by 3% in the fiscal year 2020–2021. In 2021, Liberia was 173 out of 186 countries on the Human Development Index, with a score of 0.465. Liberia has a national poverty rate of 50.9% and a Multidimensional Poverty Index (MPI) of 0.259. Around 71.2% of Liberians are living in multidimensional poverty. Of those, 33.2% are extremely poor, and 20.4% are at risk of falling back into poverty. Before the coronavirus disease 2019 outbreak, Liberia was one of the top 10 countries for a rapidly lowering (MPI).

The formal sector is not well developed and only offers about 20% of jobs, with many people working in the informal sector. About 52% of women and 75% of men in Liberia can read and write. In Liberia, about 31% of women between the ages of 15 years and 49 years do not have an education, while just 13% of men have.^1^

Access to safe water sources is 84%, and to sanitation is 47%. In Liberia, 42% of people defecate in the open, with 61% of people doing so in rural areas and 23% in urban areas. About 18% of people in Liberia do not have enough food, and 2% of those people are very food insecure.^1,2^

## Levels of healthcare and system organisation

The health system of Liberia is founded on the ideas of primary health care (PHC). It consists of three tiers: primary, secondary, and tertiary, which align with the national health policy and strategic plan.^2^

The composition of the different tiers is as follows:

- Primary Care: community health services and clinics.
- Secondary Care: health centres, district, and county hospitals.
- Tertiary Care: regional and referral hospitals.^2^

### Primary health care: Community health services and clinics

Community health assistants (CHAs), community health promoters (CHPs), trained traditional midwives (TTMs), and community animal health workers (CAHWs) are all part of the community health workforce. The community chooses health workers, and then the Ministry of Health trains them to provide community health care packages. The National Community Health Services Policy 2022–2032 sets the rules for how to choose people.

The CHAs help people who live more than 5 km or an hour’s walk from the nearest health facility, while the CHPs help those who live within 5 km of a health facility. The Community Health Policy recommends the following ratios: one CHA for 40–60 households (up to 350 people); one CHP for 60–80 households (up to 500 people); and one TTM for 20–30 homes, which can hold up to 150–200 people.

The CHAs provide preventative and promotional services, as well as other curative services, including diagnosing and treating malaria. They focus on children up to the age of 11 years. The CHAs connect the community to the health system by providing services in the community, making it easier for people and groups to get health services, and teach community members about health issues.

The CHPs provide services that promote health and prevent disease while linking people to care at the facilities. They provide similar services to the CHAs, but do not provide curative services like integrated Community Case Management (iCCM), medroxyprogesterone acetate injectable contraceptive, and other injectable therapies. Community Health Promoters are another link between the health system and the community.^2^

### The health clinic

The health clinic serves a population of between 3500 and 12 000 people and offers outreach services to parts of the community that are more than 5 km away. Clinics must be open for at least 8 h a weekday, and their doctors, nurses and midwives must be able to provide basic emergency obstetric and neonatal care (BEmONC). The clinics are expected to attend to all diseases and problems across all ages but refer those that are beyond their capacity, skills and knowledge.

### Health workforce challenges in Liberia

The health workforce density prior to the outbreak of Ebola was less than 3.7 per 10 000 people. It is estimated that there will be a shortage of 2700 healthcare personnel by the year 2030. Two-thirds of the population are in rural regions, compared to just one-third of physicians and nurses. Training platforms, mentorship, and career assistance are the primary focusses of attempts to retain employees in remote rural areas.^4^

### Family medicine development

Family medicine (FM) in Liberia is an emerging specialty, critical for PHC and universal health coverage (UHC). It emphasises first contact, person-centred, and comprehensive care, across the burden of disease and including essential public health functions. Family Medicine in Liberia differs from high-income countries by emphasising procedural skills in surgery, obstetrics and anaesthetics, due to limited specialist availability.^4^

Family physicians (FPs) in Liberia play a crucial and multi-level role, from outpatient clinics in teaching hospitals to primary care in rural and underserved areas. They provide clinical care, mentorship, management, and leadership across all the three tiers, with a strong emphasis on strengthening primary care and addressing workforce shortages.^4,5^ Currently, there are 30 FPs on the national register. Nineteen are employed in the public sector, two in the private sector and one in the military. The rest are general practitioners and FPs in training.

### Postgraduate training

The Family Medicine Specialty Training Program (FMSTP) is a postgraduate initiative provided by the Liberia College of Physicians and Surgeons (LCPS), which holds regional accreditation from the West African College of Physicians (WACP). This 3-year programme adheres to WACP requirements and integrates longitudinal rural rotations into its curriculum. The programme commenced in July 2017, with its inaugural rural rotation occurring in January 2018 at the Eternal Love Winning Africa Hospital in Monrovia. Rural training was subsequently relocated to JJ Dossen Hospital in Maryland County and John F. Kennedy Memorial Medical Centre in Monrovia. By 2025, the programme *had* educated more than 20 FM specialists.

The primary aim of the FMSTP is to mitigate the deficit of healthcare practitioners in rural regions, given that most Liberian physicians are based in Monrovia. The programme’s rural rotations aim to familiarise residents with the distinct health difficulties encountered by underprivileged regions, emphasising practical patient care, community health, and mentorship. The programme attains its objectives via partnerships with entities including Partners In Health (PIH), Tubman University, Eternal Love Winning Africa (ELWA) Hospital, John F. Kennedy Memorial Medical Centre, and many establishments managed by the local Ministry of Health. The programme’s effectiveness is consistently assessed via resident comments, faculty evaluations, and accreditation review outcomes.^4,6^

### Eternal Love Winning Africa

This 5-year programme (3-years for Membership or Part I training and 2-years for Fellowship or Part II training) adheres to WACP requirements and integrates longitudinal rural rotations into its curriculum. The programme commenced in July 2017, with its inaugural rural rotation occurring in January 2018 at the Eternal Love Winning Africa Hospital in Monrovia. Rural training was subsequently relocated to James Jenkin Dossen Hospital in Harper City in Maryland County, and a few rotations in other specialties such as psychiatric at E.S. Grant Mental Health Hospital in Monrovia and pathology at the John F. Kennedy Memorial Medical Centre in Monrovia. By 2025, the programme had educated more than 20 FM specialists. Prior to commencement of FMRT programme, there was no known formal registry of number of practicing FPs in Liberia.^7^

### Society of Family Physicians of Liberia

The Society of Family Physicians is an umbrella organisation that brings together General Practitioners and Family Physicians working in the country to collaborate on the challenges affecting them and look at ways of improving FM training in the country. It subscribes to World Organization of National Colleges, Academies and Academic Associations of General Practitioners/Family Physicians (WONCA), and as a member organisation, the leadership participates in regular meetings. The membership is open to all General Practitioners and Family Physicians. It is registered under the laws of Liberia as an autonomous entity.^4^

## Conclusion

Liberia has made significant strides in establishing a PHC system and advancing FM as a vital specialty in the country. Though the integration of FM into Liberia’s health system is ongoing, it is increasingly recognised as essential for achieving UHC and enhancing health outcomes, particularly in rural areas facing workforce shortages. Future efforts focus on expanding the FMSTP to produce more graduates and improve rural training, alongside initiatives aimed at supporting and retaining health workers through mentorship and community engagement. Primary care delivery will emphasise comprehensive, person-centred care addressing both communicable and non-communicable diseases while engaging national and international partnerships to enhance training and resources. Liberia’s experiences demonstrate the importance of context-specific training, long-term rural rotations, collaborative partnerships, and continuous evaluation, offering valuable lessons for other low-resource settings striving for resilient healthcare systems.
